# Word frequency and cognitive effort in turns-at-talk: turn structure affects processing load in natural conversation

**DOI:** 10.3389/fpsyg.2024.1208029

**Published:** 2024-06-05

**Authors:** Christoph Rühlemann, Mathias Barthel

**Affiliations:** ^1^University of Freiburg, Freiburg, Germany; ^2^Pragmatics Department, Leibniz Institute for the German Language (IDS), Mannheim, Germany

**Keywords:** conversation, corpora, word frequencies, turn-taking, turn structure, processing load, speech production, pupillometry

## Abstract

Frequency distributions are known to widely affect psycholinguistic processes. The effects of word frequency in turns-at-talk, the nucleus of social action in conversation, have, by contrast, been largely neglected. This study probes into this gap by applying corpus-linguistic methods on the conversational component of the British National Corpus (BNC) and the Freiburg Multimodal Interaction Corpus (FreMIC). The latter includes continuous pupil size measures of participants of the recorded conversations, allowing for a systematic investigation of patterns in the contained speech and language on the one hand and their relation to concurrent processing costs they may incur in speakers and recipients on the other hand. We test a first hypothesis in this vein, analyzing whether word frequency distributions within turns-at-talk are correlated with interlocutors' processing effort during the production and reception of these turns. Turns are found to generally show a regular distribution pattern of word frequency, with highly frequent words in turn-initial positions, mid-range frequency words in turn-medial positions, and low-frequency words in turn-final positions. Speakers' pupil size is found to tend to increase during the course of a turn at talk, reaching a climax toward the turn end. Notably, the observed decrease in word frequency within turns is inversely correlated with the observed increase in pupil size in speakers, but not in recipients, with steeper decreases in word frequency going along with steeper increases in pupil size in speakers. We discuss the implications of these findings for theories of speech processing, turn structure, and information packaging. Crucially, we propose that the intensification of processing effort in speakers during a turn at talk is owed to an informational climax, which entails a progression from high-frequency, low-information words through intermediate levels to low-frequency, high-information words. At least in English conversation, interlocutors seem to make use of this pattern as one way to achieve efficiency in conversational interaction, creating a regularly recurring distribution of processing load across speaking turns, which aids smooth turn transitions, content prediction, and effective information transfer.

## 1 Introduction

Corpus linguists are well versed in mining and statistically evaluating word frequencies, which are key to computing collocations, colligations, collostructions, n-grams, semantic prosodies, and semantic associations. They do not need to be convinced that “strictly speaking at last, the only thing corpora can provide is information on frequencies” (Gries, 2009, p. 11). What frequencies “mean” in the context of lexis-lexis and text-lexis co-occurrence phenomena is well researched (e.g., Hoey, 2005). The relevance of frequencies for turn-taking in social interaction has, by contrast, been neglected so far, even though taking turns-at-talk in face-to-face conversation is the prime ecological niche for language use, “where the bulk of language usage occurs” (Holler and Levinson, 2019, p. 639), and the turn is “very likely the basic form of organization for talk-in-interaction” (Schegloff, 2001, p. 230).

Frequency effects are claimed to be “all-pervasive” (Kreyer, 2014). They can be found “in the processing of phonology, phontactics, reading, spelling, lexis, morphosyntax, formulaic language, language comprehension, grammaticality, sentence production, and syntax” (Ellis, 2002, p. 143). According to research in information theory, speakers have access to word frequency and collocation frequency information stored in the mental lexicon (e.g., Jaeger, 2010; Seyfarth, 2014). This knowledge is available not only to adults. Even sixth-graders can estimate the relative frequencies of words with very high degrees of accuracy (Shapiro, 1969), which is one of the reasons why frequency processing is assumed to be automatic, other reasons being that it is fast and seemingly effortless (Hasher and Chromiak, 1977).

Psycholinguistic research has shown high word frequency to speed up processing in both comprehension and production. For example, in lexical decision experiments, decisions on frequent words are faster than on infrequent words (Balota et al., 2007). Also, turns consisting of higher frequency words are comprehended and produced faster, thus reducing turn-transition times (Roberts et al., 2015, p. 3). Frequency information on collocational patterning helps in resolving ambiguities both in lexis (e.g., *well*'s multiple syntactic and pragmatic functions; cf. Rühlemann and Gries, 2021) and syntax (as in *The spy saw the cop with the binoculars;* cf. Ellis, 2002). Similarly, a long-standing tradition of research starting with Oldfield and Wingfield (1965) has established that word frequency has a significant effect on production latency. For example, in Jescheniak and Levelt's (1994) picture naming experiment, high-frequency words with a frequency above 60 per million were articulated significantly faster than low-frequency words with a frequency below twelve per million (cf. also Levelt et al., 1999; Indefrey and Levelt, 2004). A similar frequency effect has been observed on the sublexical level, with high-frequency syllables being produced significantly faster (independent of the word frequency effect; Levelt and Wheeldon, 1994)^1^ and with greater error-resistance under adverse conditions (as in aphasic speech or tongue twisters) (Aichert and Ziegler, 2004; Wulfert et al., 2022).

The notion that word frequency is structured rather than uniform or haphazard has been famously introduced by Zipf (1935/1965). Based on the observation that few high-frequency words account for most of the tokens in text, he posited what came to be known as Zipf's law, suggesting that a word's total frequency in a given corpus is inversely proportional to its rank in the frequency table, with the most frequent word occurring approximately twice as often as the second most frequent word, three times as often as the third most frequent word, and so forth. While this law states an observable statistical regularity [but see Piantadosi's (2014) critical review] and does not yet incorporate any consideration of *language use*, Zipf (1949/1965) also noted a fundamental relation between a word's frequency and its length, with highly frequent words (such as pronouns or auxiliaries) being of short phonetic size and very infrequent words tending to be long in phonetic size. He attributed this correlation to an “economy of words” (Zipf, 1949/1965, p. 19) operating in the service of what he termed the Principle of Least Effort. This principle, which is claimed to govern every individual's entire behavior, suggests that we seek to minimize our *probable average work-expenditure over time*. The phonetic brevity of high frequency words thus reduces the expenditure required given their commonness.

Research on corpora of talk in interaction recently proposed the notion that the distribution of word frequencies within turns is structured. Rühlemann (2020a) shows that turns-at-talk overwhelmingly start on high-frequency items, be they pronouns such as *I* (the most common word in conversation), or what has been termed “inserts”, including most prominently interjections such as *oh, well*, or *yeah* (cf. also Tao, 2003). Moreover, Yu et al. (2016) found a three-step staircase-shaped distribution for mean frequencies in (written) English sentences, starting with a high-frequency word, dropping to intermediate-frequency words in sentence internal positions, before dropping again to a low-frequency word in sentence-final position. This finding has been replicated in Klafka and Yurovsky (2021) and also emerged in a descriptive analysis of 7 to 12-word turns contained in the conversational component of the British National Corpus (Rühlemann, 2020a).

With frequency notoriously affecting language processing on numerous linguistic levels, it is surprising that the effects of frequency on the processing of turns-at-talk has received very little scholarly attention. This study aims to fill this gap by investigating the structure of frequency in turns drawing on data from two corpora: the British National Corpus (BNC) and the Freiburg Multimodal Interaction Corpus (FreMIC; see *Section 2.1 Corpora*). The FreMIC, a novel corpus (Rühlemann and Ptak, 2023), holds information about interlocutors' pupil sizes that were recorded alongside the conversations. While pupil size is affected by a number of factors, including lighting conditions, drug consumption, pathological states, and emotional arousal, it is known to also reflect processing intensity (Beatty, 1982; Beatty and Lucero-Wagoner, 2000; Sirois and Brisson, 2014; cf. references in Laeng et al., 2012, p. 18) and has been successfully used in experimental psycholinguistic studies as a reliable indicator of processing load during language comprehension (e.g., Just and Carpenter, 1993; Kuchinke et al., 2007; Engelhardt et al., 2010; Schmidtke, 2014; Koch and Janse, 2016; Tromp et al., 2016) and production (Papesh and Goldinger, 2012; Sevilla et al., 2014; Lõo et al., 2016; Sauppe, 2017; Barthel and Sauppe, 2019). These studies consistently find that speakers' and comprehenders' pupils dilate more in conditions of increased processing effort as compared to conditions of reduced processing effort. This finding holds not only for monolog, but also for dialogical tasks, as Barthel and Sauppe (2019) found speakers who had to respond to their interlocutor to show increased pupil sizes in the vicinity of turn-transitions when they were planning their response in overlap with the incoming turn as compared to when they planned their response in silence after the incoming turn.

Given the availability of pupillometric data in FreMIC, this study breaks new ground as it uses pupillometry in a corpus of unconstrained conversation. Based on the aforementioned previous findings in laboratory conditions on the effects of word frequency on language processing on the one hand, and on pupil size as an indicator of processing load on the other hand, we hypothesize that speakers' and/or recipients' pupil sizes in the FreMIC corpus will be affected by the frequencies of the words contained in the turns under investigation. As increased processing load goes along with increased pupil dilation, we expect decreasing word frequencies in the course of a given turn to cause pupil dilation to increase, while an increase in word frequencies within a given turn would be expected to lead to a decrease in pupil dilations. Since frequencies within turns have been found to tend to be ordered in a decreasing, anticlimactic way, we expect the former case to be the default in the analyzed corpus.

## 2 Methods

### 2.1 Corpora

The data underlying the analyses in this paper come from two English-language corpora: (i) the conversational subcorpus of the British National Corpus (BNC-C; cf. Hoffmann et al., 2008) and (ii) the Freiburg Multimodal Interaction Corpus (FreMIC; cf. Rühlemann and Ptak, 2023).

The BNC-C, on the one hand, is a widely used speech corpus in linguistic research. It consists of ca. 4.2 million words uttered in casual conversations between friends and family, most of them face-to-face, recorded via portable audiotapes. The data are transcribed orthographically and Part-of-Speech tagged using the *CLAWS-5* tag set, which distinguishes 70 Part-of-Speech categories.^2^ The accuracy rate for CLAWS-5 taggings is 98.5% (Leech et al., 1994).

FreMIC, on the other hand, is a novel corpus, both in the sense that it is new and, at the time of writing, still under construction, and in the sense that it holds information of a breadth and level of detail not commonly seen in linguistic corpora.^3^ FreMIC is a multimodal corpus of unscripted conversation in English.^4^ At the time of writing, FreMIC comprises (i) ca. 30 h of video-recordings of 18 conversations containing 210,176 words in 31,935 turns transcribed and annotated in detail^5^ and (ii) large streams of automatically generated multimodal data. All conversations are annotated and transcribed in ELAN (Wittenburg et al., 2006). The transcriptions follow both orthographic and conversation-analytic conventions (e.g., Jefferson, 2004) to render verbal content and interactionally relevant details of sequencing (e.g., overlap, latching), temporal aspects (pauses, acceleration/deceleration), phonological aspects (e.g., intensity, pitch, stretching, truncation, voice quality), and laughter. Transcriptions are organized around inter-pausal units (IPUs), i.e., annotations are separated when a speaker pauses for more than 180 ms. This threshold reflects the human threshold for detection of acoustic silences, which lies between 120 and 200 ms (Walker and Trimboli, 1982; Heldner, 2011) and it facilitates comparability with related studies (e.g., Levinson and Torreira, 2015; Roberts et al., 2015). The onsets and offsets of the IPUs were determined through inspection of waveforms and spectrograms using Praat (v6.1; Boersma and Weenink, 2015). All orthographic transcripts in FreMIC were Part-of-Speech tagged using the CLAWS web tagger (Garside and Smith, 1997) and its *c7* tag set (http://ucrel-api.lancaster.ac.uk/claws/free.html).^6^ The *c7* tag set is more fine-grained than the *c5* tag set underlying the BNC-C data in that it provides many more subcategories; the total number of PoS categories in the *c7* tag set is 138 (almost twice the number of the *c5* tag set).^7^ The accuracy rate for the *c7* tag set is 96–97% (Paul Rayson, personal communication). One of the advantages of PoS-tagging speech is that it allows for the computation of turn size based on the number of grammatical words, i.e., even in contracted forms, the underlying grammatical words are recognized, tagged, and counted separately. So, for example, the phrase “I'm gonna” is tagged “I_PPIS1 ‘m_VBM' gon_VVGK na_TO” resulting in four rather than two words.

### 2.2 Participants

Fourty-one individual participants were recruited to contribute to one or more of the 18 recorded conversations. Participants were mainly students at Albert-Ludwigs-University Freiburg as well as their friends and relatives [17 male, 21 female, 3 diverse/NA; mean age = 26 years (SD = 5.7 years)]. Most participants' first language was English (6 British, 24 American). All participants had normal or corrected to normal vision and hearing. Before the start of the recording, participants gave their informed consent about the use of the recorded data, signing their individual choices as to which of their data can be used and for what specific purposes. They received a compensation of 15 € for each recording.

### 2.3 Procedure

Recordings were made in dyadic and triadic settings using one room camera and one centrally placed scene microphone. Participants were seated in an F-formation (Kendon, 1973) enabling them to establish eye contact, hear each other clearly, and engage in nonverbal cues. Participants in dyads were seated vis-à-vis each other, with the room camera capturing both participants from the side. Participants in triads were seated in an equilateral triangle, with the room camera frontally capturing one of the participants and the other two from the side. The participants were instructed that they were free to talk about whatever they liked for about 30–45 min until the recording would be stopped.

Participants wore Ergoneers eyetracking devices (Dikablis Glasses 3), which recorded the visual field of each participant plus the direction of participants' gazes as well as their pupil sizes (with pupil size values stored in pixels at an average frequency of 60 Hz). Participants were informed that the eyetrackers were used to record their gaze behavior during conversation. To calibrate the devices, participants were instructed to look clockwise at the corners of a rectangular sheet with numbers in each corner (from 1 to 4) while the instructor checked with D-Lab (the Ergoneers eyetracking software) whether the participant's gaze indicator actually hit that corner. In a second step, the instructor would mention the corner numbers in random order to see if the participant's glance indicator matched these numbers.

Since pupillometric data were collected in casual conversations with limited provisions to fully control environmental lighting conditions, we are aware that there is likely considerable noise in the pupil data. Given the large number of conversational turns in the corpus, it is still possible to detect regularities in pupil size changes in interlocutors (see *Section 3 Results*).

### 2.4 Data pre-processing

All ‘'turns”^8^ ranging between 3 and 25 words in length were selected from both corpora. The resulting subsets contain 291,447 turns (61%) of the BNC-C and 18,095 turns (57%) of FreMIC. Each word token in these turns was assigned its subset-internal token frequency calculated on the basis of word-tag combinations (cf. *Section 2.1*). In preparation of the descriptive analysis, mean frequencies were computed for each turn size and each word position therein. For the mixed-effects regression models, normalized word frequencies (per thousand words) were computed for each word type.

Measured pupil sizes were averaged over both pupils (e.g., Barthel and Sauppe, 2019). To account for blinks, pupil area values were linearly interpolated in batches of 600 observations each. Pupil size values were baselined to 0 at turn start, i.e., for each turn and participant, the first pupil area datum of a turn was subtracted from all following values within that turn. Pupil size values were then divided into equally sized bins, with the number of bins determined by the number of words in the respective turn (e.g., 100 pupil data points of a particular participant contained in a 4-word turn were divided into four bins of 25 pupil data points each). For each bin (i.e., for each word *w1, w2, w3*, etc. in the turn) the mean pupil size was calculated for each participant.^9^ On average, each bin contained 15.79 pupil size observations (median = 14.67; SD = 5.84). For all mixed-effects models, word position in turns was normalized by dividing each position minus 1 by the number of words present in the turn minus 1, so that word positions within turns are always quantified between 0 (first word) and 1 (last word), irrespective of turn size. Turn size was centered before being used as a control variable in the mixed-effect models.

### 2.5 Statistical analyses

Statistical analyses on frequency distributions and pupil size changes were conducted in R (v4.3.0; R Core Team, 2023). Descriptive analyses of normalized word frequency distributions were based on visual inspection of plotted mean word frequencies per word position, both in the BNC-C and the FreMIC corpora. Mixed effects regression models on the FreMIC corpus data were built using the R-package *lme4* (Bates et al., 2015). Statistical significance of single predictors in the form of *p*-values were obtained with the R-package *lmerTest* (Kuznetsova et al., 2017). Participant role (speaker vs. recipient) was contrast coded. Word class (noun vs. function word vs. insert) was deviation coded. Where applicable, orthogonal polynomial predictors of dependent variables were computed using the *poly()* function from the R-package *stats*. Model selection was run by first removing any non-significant (*p* > 0.05) interactions of main predictors of interest with control variables (like turn size), and in a following step adding a quadratic predictor (allowing for a curved fit) of a factor of main interest to a model containing the linear predictor, keeping the quadratic term if it was statistically significant below a threshold of *p* < 0.05. In that case, a cubic predictor (allowing for an S-shaped fit) was added to the model containing the linear and the quadratic predictor and kept if it was significant below the same threshold. In that case, the next higher level polynomial was included in the model and so on.

A mixed model was built, modeling the development of word frequencies within the utterances, i.e., across word positions in the utterances (see *Section 2.4 Data Pre-Processing*). The position of each of the words within an utterance and the scaled size of the utterance containing it as well as their interaction were included as fixed effects. Random intercepts by participant and by conversation were added as random effects.

A regression model was built to model the percentages of occurrence of different word classes within the BNC-C utterances across the positions within turns. As percentages were aggregated over turns and positions therein, participant and file information could not be obtained and were therefore not modeled.

A related model was computed to model the percentage of occurrence of hapax nouns (i.e., nouns that occur just once in the corpus) within the BNC-C utterances across the positions within turns.

Another mixed model was built modeling the development of participants' pupil size within utterances, i.e., as an utterance unfolds. As the dependent variable, pupil sizes within each utterance were binned into as many even sized bins as there are words in the respective utterance (see *Section 2.4 Data Pre-Processing*). The position of each bin within an utterance and the role of the participants in each utterance as well as their interaction plus the scaled size of the utterance were included as fixed effects. Random intercepts by participant and by conversation were added as random effects.

A forth mixed model was built to assess the correlation of the trends in the frequencies of words in the utterances in the data set and the trends in pupil size changes in the participants. For that model, linear slope coefficients in both word frequencies and pupil sizes of each participant were computed for each utterance using the *lm()* function from the *stats* package. The resulting trends in pupil size in each participant in each utterance were then modeled, using the previously computed trends in word frequencies in each utterance and the role of the respective participant in that utterance (speaker vs. recipient) as well as their interaction as fixed effects plus the scaled size of the utterance as an additional control variable in the fixed effects structure. Random intercepts by speaker and by conversation were included as random effects.

To assess the significance of simple effects causing significant interactions, post-hoc tests were based on *F*-tests comparing estimated marginal means of factor levels (Searle et al., 1980) that were conducted using the *R* package *emmeans* (v1.8.0; Lenth, 2022).

## 3 Results

### 3.1 Frequency distributions across utterances

**Figure 1:**
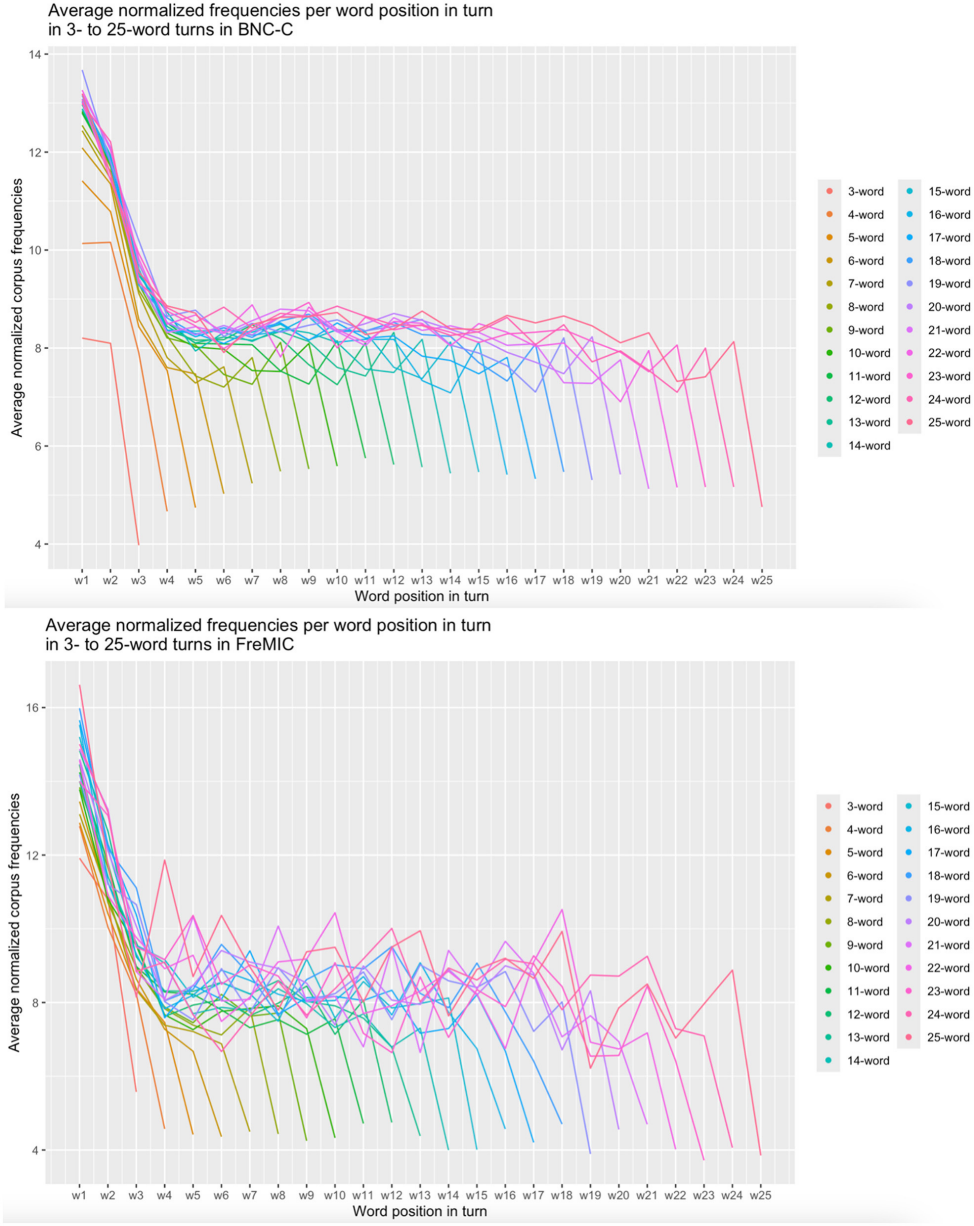
Average absolute corpus frequencies per turn position for 3- to 25-word turns in BNC-C (**upper panel**; n = 291,447 turns in 153 files) and in FreMIC (**lower panel**; n = 18,095 turns in 18 conversations).

The two line graphs in Figure 1 reveal a striking pattern: Average normalized corpus frequencies start very high in turn-initial position (*w1*), then drop in turn-medial positions, then level out until the last position in the turn, where they drop again steeply. This pattern emerges very clearly and with little variation in the BNC-C data and also, though with more variation, in the FreMIC data. The pattern is thus exactly the same three-step staircase-shaped distribution that Yu et al. (2016) found for mean frequencies across different positions in written English sentences (see also Klafka and Yurovsky, 2021).

**Figure 2:**
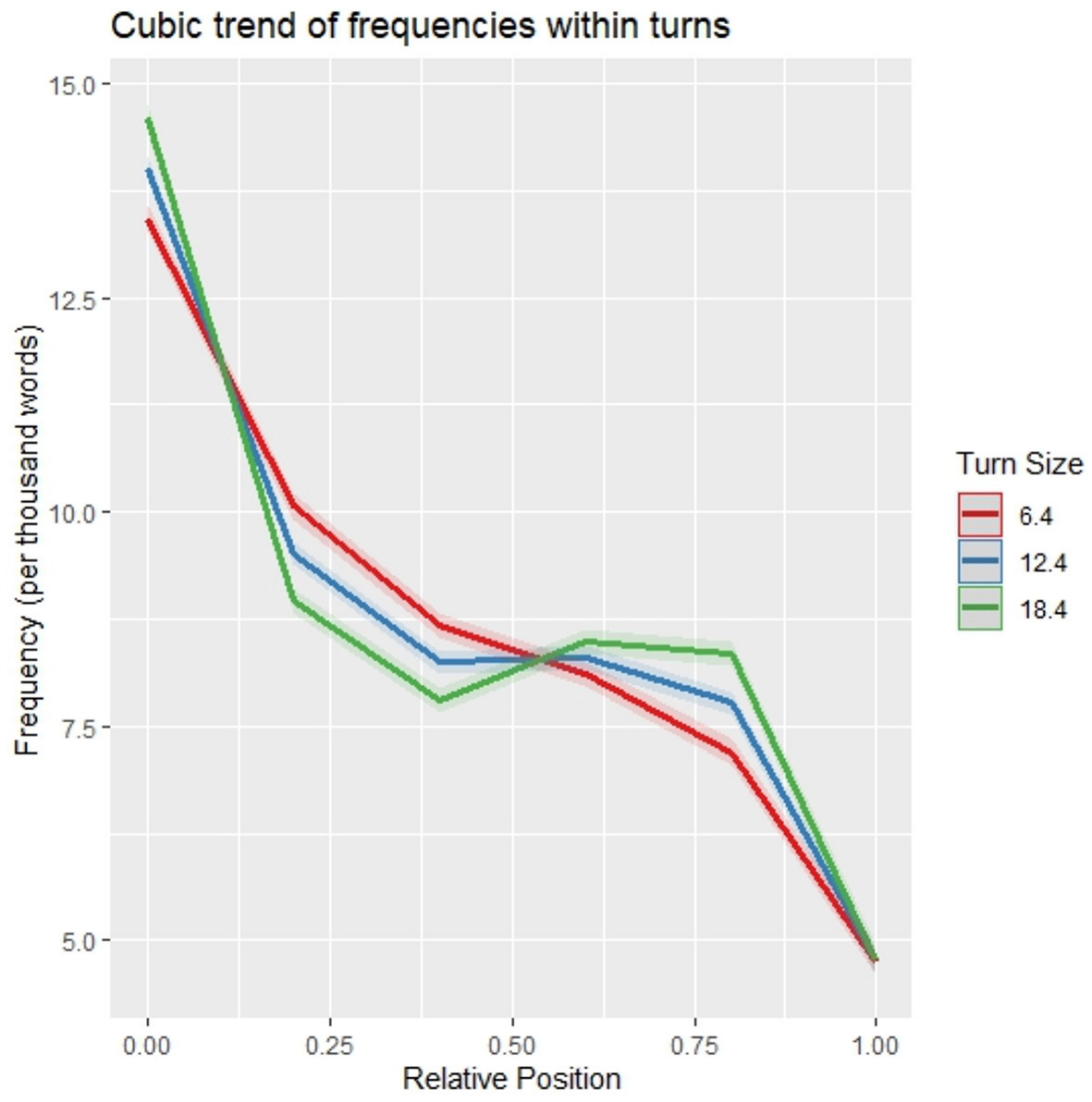
Modeled frequencies within utterances. For plotting only, unscaled turn sizes and uncentered relative word positions were used. Shaded areas indicate one standard deviation of the mean.

To assess the statistical reliability of the visually observed pattern of frequency distribution within utterances, normalized word frequencies were modeled with a third-order polynomial of position and utterance size as well as their interaction as fixed effects (following the model selection steps described in *Section 2.5 Statistical Analyses*) and random intercepts by speaker and by conversation as random effects (see Table 1 for model output). All three polynomial terms of position were significant (p < 0.001), showing that the distribution pattern of word frequencies within utterances is well-described by an S-curve with steep decreases of frequencies near the beginning and end of utterances (Figure 2).^10^

**Table 1:** Output of linear mixed-effects regression model on word frequencies within utterances.

**Fixed effects**				
	β	***SE***	***t***	***p***
Intercept	8.716	0.12	67.447	
Position	−883.8	11.59	−76.283	< 0.001 ^***^
Position^2^	174.6	11.43	15.273	< 0.001^***^
Position^3^	−391.6	11.58	−33.809	< 0.001^***^
Size	0.041	0.02	1.480	0.139
Position: Size	51.05	11.42	4.472	< 0.001^***^
Position^2^: Size	−66.32	11.07	−5.984	< 0.001^***^
Position^3^: Size	−152.3	11.57	−13.155	< 0.001^***^

Formula = f_perThousand ~ poly(position_rel, degree = 3) ^*^utteranceSize_scaled + (1 | speaker) + (1 | conversation). Asterisks indicate levels of significance: ^***^indicates *p* < .001.

**Figure 3:**
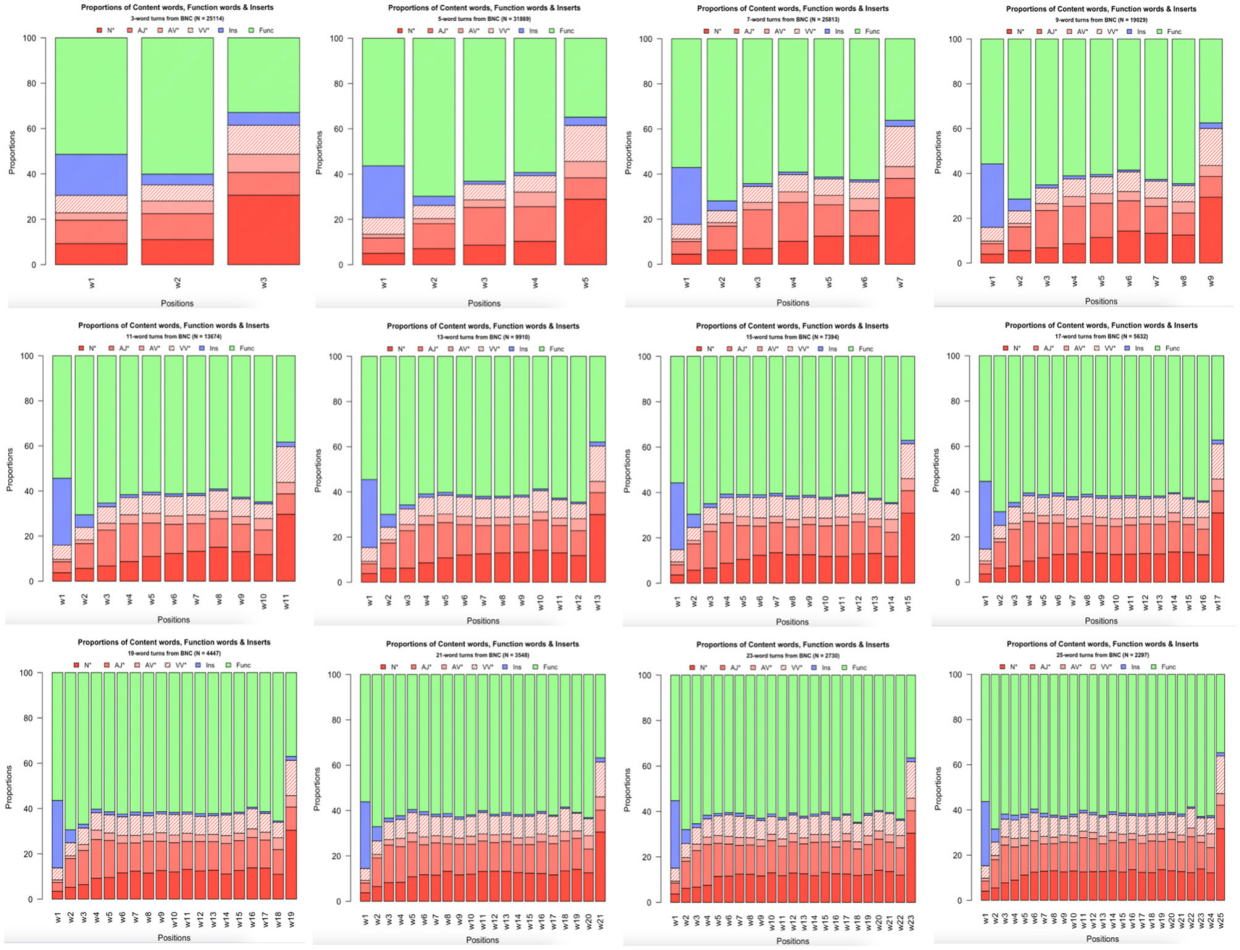
Percentages of content words, function words, and inserts in 3-, 5-, 7-, 9-, 11-, 13-, 15-, 17-, 19-, 21-, 23-, and 25-word turns in BNC-C.

To analyze the underlying structures of the attested regularities in frequency distribution within turns-at-talk, we categorized all words in the BNC-C depending on their PoS tag as either a type of (i) content word—that is, as adjectives, adverbs, lexical verbs (but not auxiliary and modal verbs), and nouns (cf. Biber et al., 1999)—, (ii) insert (tokens tagged ITJ as well as *well* and *so* (which are mis-tagged as adverbs in the BNC-C),^11^ or (iii) function word (all remaining tokens). Figure 3 depicts the percentages of these word class tokens per position within turns in a number of representative turn sizes. Nouns in particular show an increase in percentages at the end of turns. In the turn sizes investigated (3-, 5-, 7-, 9-, 11-, 13-, 15-, 17-, 19-, 21-, 23-, and 25-word turns), they have a mean proportion of 4.20% in turn-initial position (median = 3.84%; range = [2.80%; 9.27%]; SD = 1.29), and a mean percentage of 29.8% in turn-final position (median = 29.9; range = [26.9%; 31.7%]; SD = 0.97). Inserts, by contrast, are found to be most frequent in turn-initial positions, drastically declining in percentage immediately afterwards. Function words show the inverse distribution to nouns, as their percentages rise after a relatively low start in the turn-first position, stay rather constant throughout the turn until they show a sudden drop in turn-final position.

**Figure 4:**
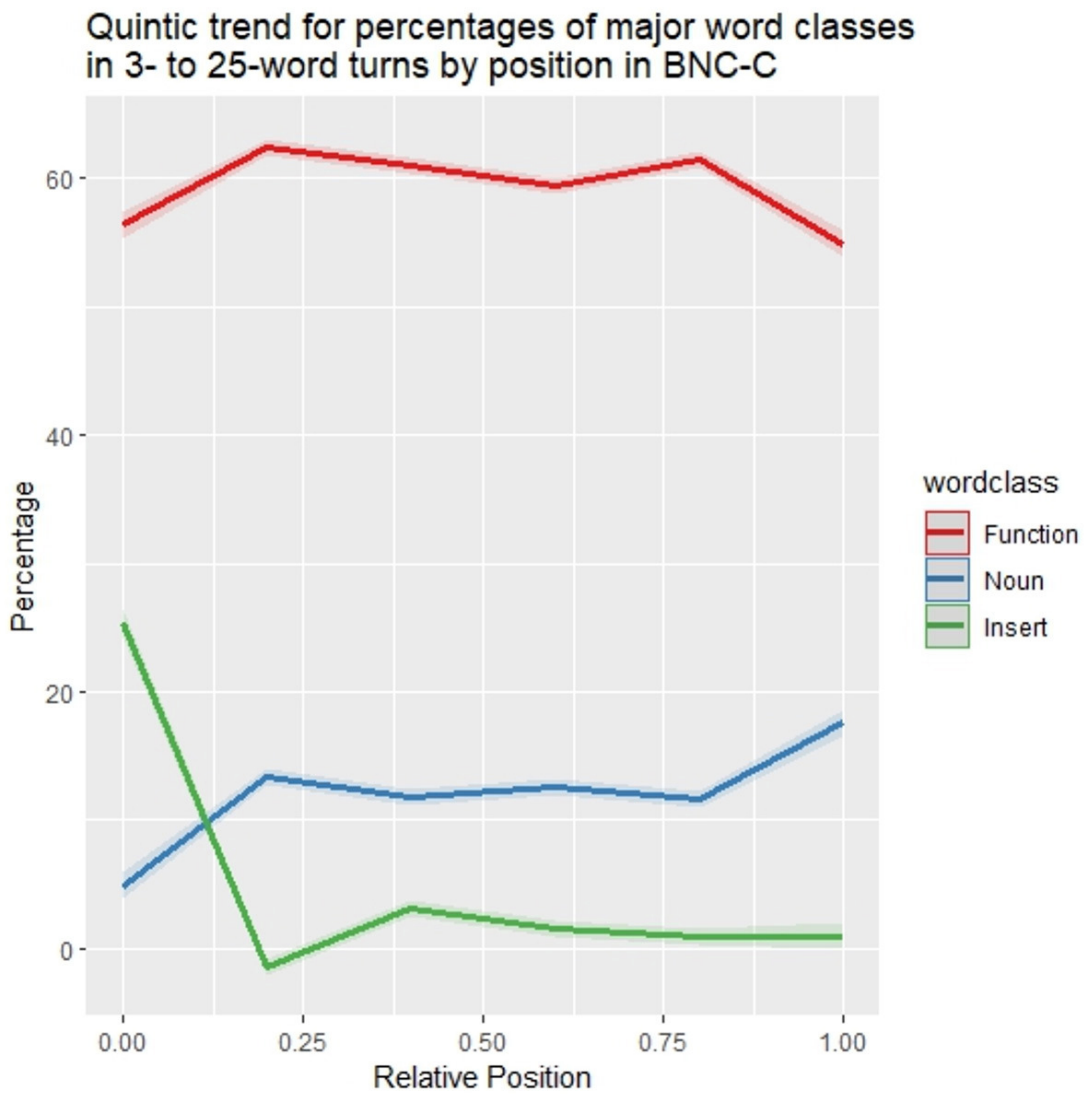
Quintic trends for percentages of nouns, function words, and inserts in the BNC-C by position. For plotting only, uncentered relative word positions were used. Shaded areas indicate one standard deviation of the mean.

To assess the statistical validity and generalizability of the patterns observed in Figure 3 over the whole spectrum of 3- to 25-word turns, word type percentages were modeled following the model selection procedure described in *Section 2.5* with a fifth-order polynomial of centered relative position and word class (function word vs. noun vs. insert), as well as their interaction as predictors using the *lm()* function in R (see Table 2 for model output). All five polynomial terms of position were significant (p < 0.05), showing that the distribution patterns of word class percentages within utterances is well-described by curves with four points of inflection (Figure 4). Notably, inserts show a steep decrease in percentages right after turn beginnings, with both nouns and function words rising, and nouns show a steep increase in percentages near the end of turns, with function words declining. This means that inserts have their highest proportion of occurrence in turn-initial position, nouns have their highest proportion in turn-final position.

**Table 2:** Output of linear regression model on percentages of word classes within utterances.

**Coefficients**				
	β	***SE***	***t***	***p***
Intercept	25.182	0.168	149.602	
Position	−20.429	5.231	−3.905	< 0.001^***^
Position^2^	17.119	5.231	3.272	0.001^**^
Position^3^	−12.248	5.231	−2.341	0.019^*^
Position^4^	11.326	5.231	2.165	0.030^*^
Position^5^	−13.910	5.231	−2.659	0.007^**^
Wordclass_noun	−13.083	0.238	−54.962	< 0.001^***^
Wordclass_insert	−21.812	0.238	−91.630	< 0.001^***^
Position : Wordclass_noun	64.489	7.398	8.716	< 0.001^***^
Position^2^ : Wordclass_noun	−25.663	7.398	−3.469	< 0.001^***^
Position^3^ : Wordclass_noun	65.941	7.398	8.912	< 0.001^***^
Position^4^ : Wordclass_noun	−19.931	7.398	−2.694	0.007^**^
Position^5^ : Wordclass_noun	47.873	7.398	6.471	< 0.001^***^
Position : Wordclass_insert	−72.838	7.398	−9.845	< 0.001^***^
Position^2^ : Wordclass_insert	83.322	7.398	11.262	< 0.001^***^
Position^3^ : Wordclass_insert	−85.223	7.398	−11.519	< 0.001^***^
Position^4^ : Wordclass_insert	77.780	7.398	10.513	< 0.001^***^
Position^5^ : Wordclass_insert	−47.268	7.398	−6.389	< 0.001^***^

Formula = percentage ~ poly(position_rel, degree = 5) ^*^ wordclass. Asterisks indicate levels of significance: ^*^ indicates *p* < .05; ^**^ indicates *p* < .01; ^***^ indicates *p* < .001.

**Figure 5:**
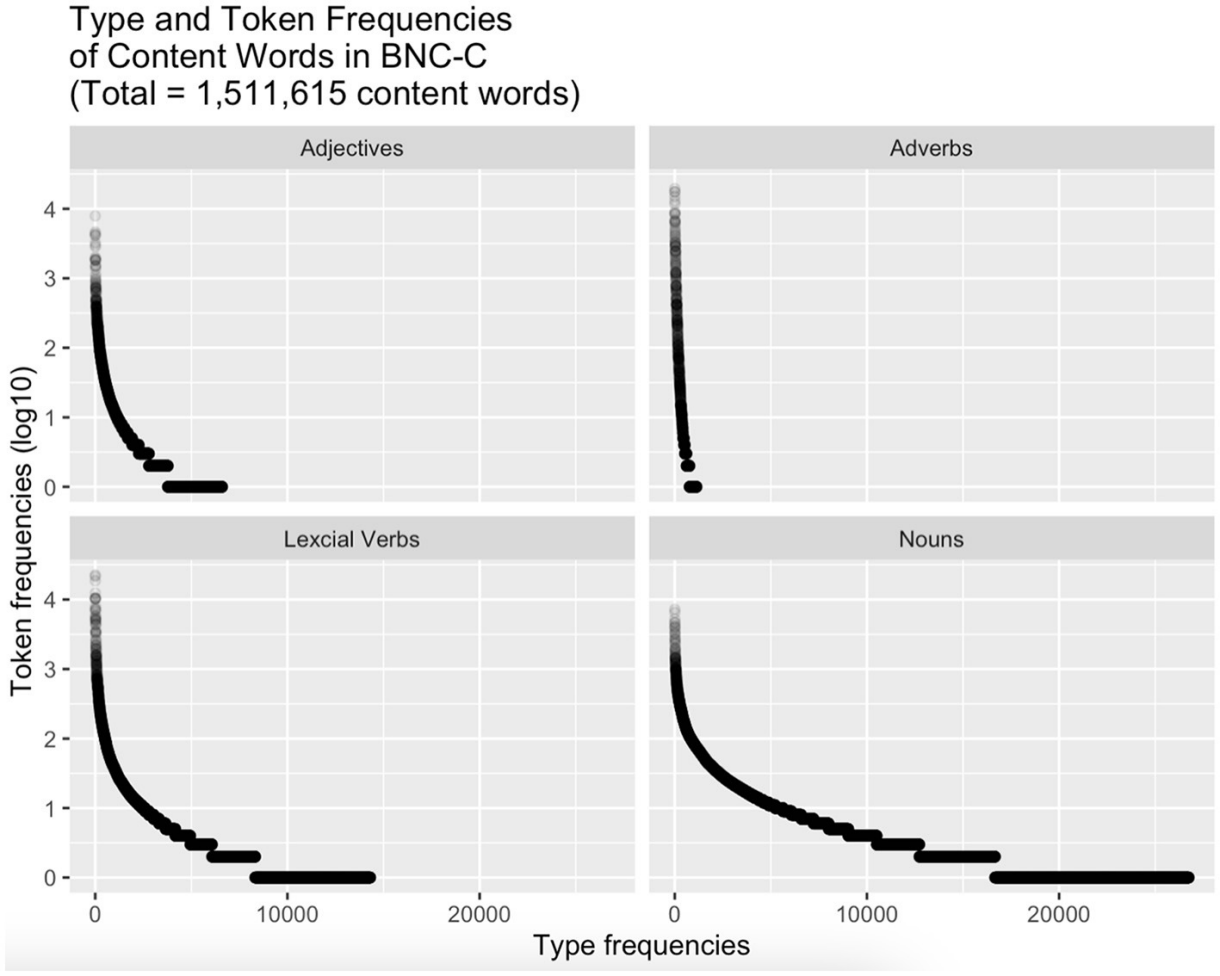
Frequency distribution of types of content words (adjectives, adverbs, lexical verbs, and nouns) in the BNC-C. Y-axis shows token frequency for each word type. X-axis shows IDs of distinct word types (i.e., the number of distinct words)^a^. ^a^Each word form is a word token; repeated occurrences of the same word form, or token, lead to larger token frequencies. One and the same word form, or token, however, is just one word type [cf. Stubbs (2002: 133 ff)].

Notably, the pattern for nouns observed in Figures 3, 4 is the *inverse* of the frequency pattern discovered in Figures 1, 2, where frequencies started out high, dropped after turn-initial positions, remaining about level on intermediate frequencies before dropping again in turn-final position. To investigate the relation of the turn-final decrease in frequencies on the one hand and the accompanying change in the distribution across word classes, Figure 5 shows the frequency distributions of the different types of content words (adjectives, adverbs, lexical verbs, and nouns).

The cascades of horizontal lines in the four content word classes get longer and longer as token frequencies go down and type frequencies go up. The cascading can be seen clearly in lexical verbs and even more clearly in nouns, but hardly in adjectives or adverbs. The cascade of central interest here is the one at the bottom of the facets, where the logged frequencies are (close to) zero: these represent hapax legomena. The size of that last cascade is negligible in adverbs, somewhat more noticeable in adjectives, again larger for lexical verbs and much longer for nouns, which are found to have by far the greatest share in hapax legomena, that is, word types that have a total frequency of just one token (i.e., a log-frequency of 0) in the whole BNC-C, accounting for 52% of all content word hapaxes.^12^

**Figure 6:**
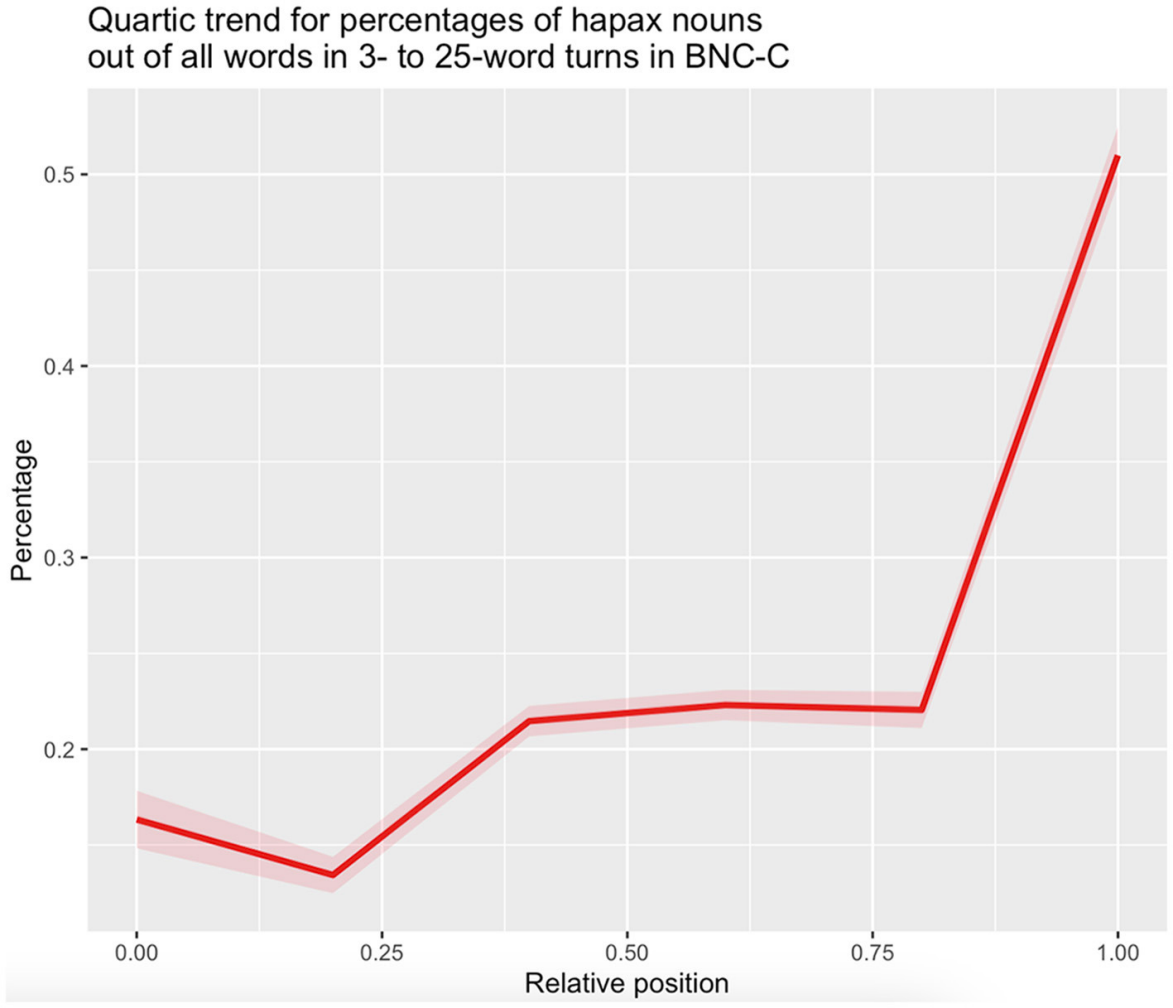
Quartic trend for percentages of hapax nouns (N = 6,129) out of all words in 3- to 25-word turns in the BNC-C by position. Shaded areas indicate one standard deviation of the mean.

In order to investigate to what extent hapax nouns contribute to the observed anticlimatic decrease in word frequency across turn positions, we modeled the percentage of hapax nouns out of all words across relative turn positions in BNC-C with a fourth-order polynomial of relative position as predictor using the *lm()* function in R (see Table 3 for model output). All four polynomial terms of position were very highly significant (p < 0.001), suggesting that the percentages of hapax nouns out of all words across positions in turns develop across three points of inflection, of which the last sets the stage for a steep rise in percentages of hapax nouns toward turn-final position (Figure 6).

**Table 3:** Output of linear regression model on percentages of hapax nouns out of all words across word positions in utterances.

**Coefficients**				
	β	***SE***	***t***	***p***
Intercept	0.224	0.004	50.421	
Position	1.390	0.079	17.438	< 0.001^***^
Position^2^	0.606	0.079	7.600	< 0.001^***^
Position^3^	0.521	0.079	6.542	< 0.001^***^
Position^4^	0.665	0.079	8.350	< 0.001^***^

Formula = percentage ~ poly(position_rel, degree = 4). Asterisks indicate levels of significance: ^***^ indicates *p* < .001.

The attested positional patterning of word frequencies might be surprising at first sight, as the subject in English would canonically be produced early in a sentence. Upon inspection, it turns out that in conversation the grammatical subject is normally not a full noun phrase but a pro-form [i.e., a function word), either a pronoun such as *I, you, he, she, it, we, they, this, that, these, those*, or the pro-form *there* (in existential clauses, like *there's someone at the door* (BNC-C: KD9)]. In 51.36% of all turns in the 3- to 25-word subset from the BNC-C (149,688 out of 291,447 turns) any of the above pro-forms occurred in a relative position ≦0.2, which is likely the subject position.^13^ Yet another 11.55% (33,649 out of 291,447 turns) did not contain a finite verb at all, indicating that these are turns without a grammatical subject. Adding in imperative turns [like *Marion, go away!* (BNC-C: KP5)], where the subject is implicit *you*, and turns where the (pronominal) subject is elided [like *Felt waxy though* (BNC-C: KBE) and *Could be* (BNC-C: KST)], it becomes evident that by far the most sizable chunk of subjects are pro-form subjects.

### 3.2 Pupil size changes across utterances

**Figure 7:**
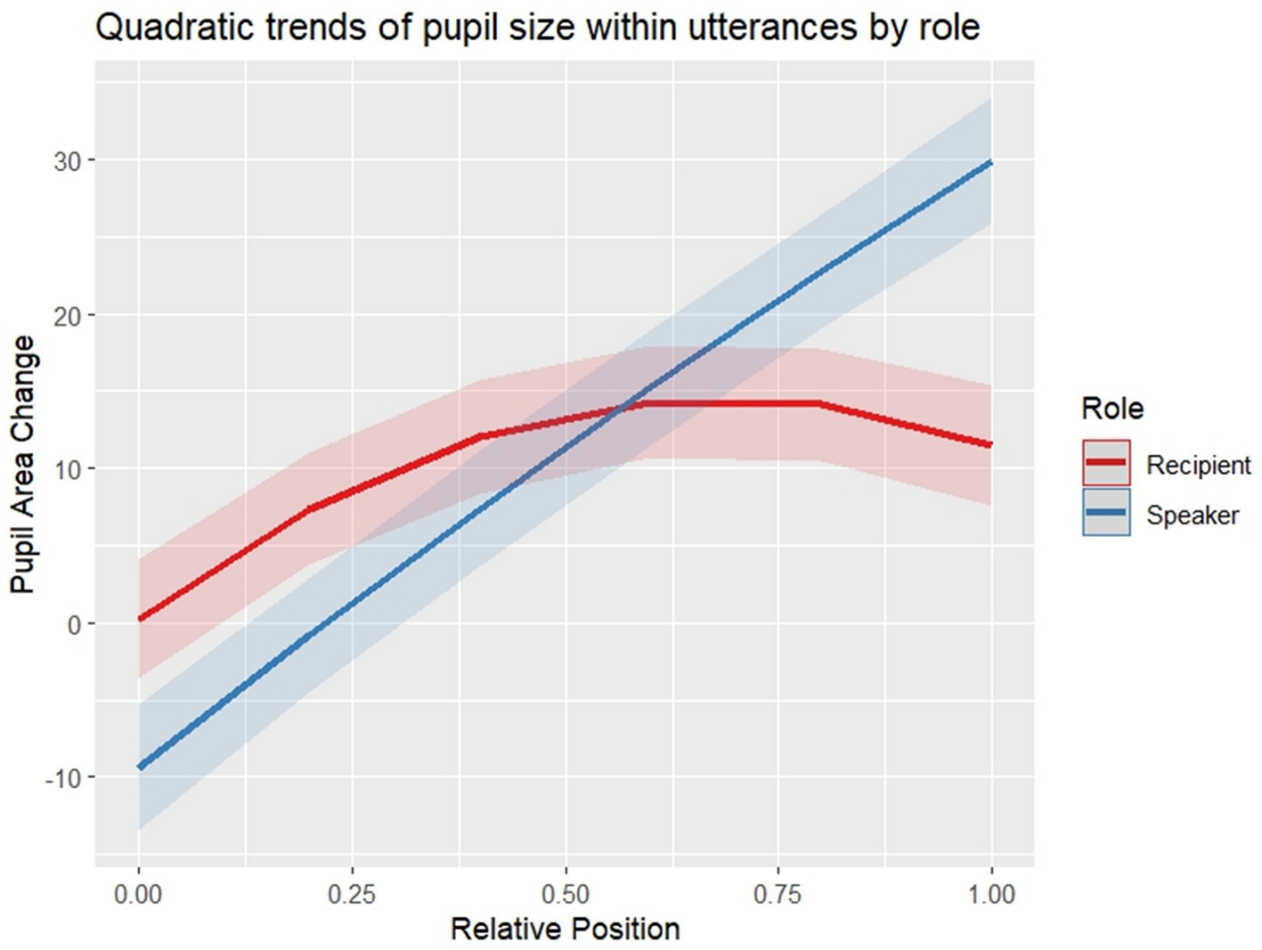
Modeled pupil sizes within utterances by role. For plotting only, uncentered relative word positions were used. Shaded areas indicate one standard deviation of the mean^a^. ^a^The integral under the curves in this figure is not 0 because pupil size is found to increase during the analyzed utterances (relative to the beginning of the utterances) and it does not return to initial size during the utterances, especially in speakers. In listeners, pupil sizes start to return already toward the end of utterances, while especially in speakers, pupil size can be expected to return during gaps between turns and possibly also during exchanges of short turns that require little planning effort (which were not modeled here).

In order to test for an increase of pupil size in participants during an utterance, and as a result of the model selection process described in *Section 2.5 Statistical Analyses*, participants' baselined pupil sizes were modeled with a second-order polynomial of position plus participant role (speaker vs. recipient) as well as their interaction and turn size as fixed effects and random intercepts of participant and conversation as random effects (see Table 4 for model output). Both main effects of the linear and the quadratic term of word position were significant (*p* < 0.001), as were their interactions with participant role (linear term: *p* < 0.001; quadratic term: *p* = 0.028), meaning that their modeled effects on the trends of speakers' and recipients' pupil size curves differ significantly, as the slope is significantly steeper in speakers than in recipients (*p* < 0.001), and the trend in recipients' pupil size change is significantly more curved than in speakers (*p*<*0.05*). *Post-hoc* tests controlling for multiple testing revealed that the linear term is significant in both speakers and recipients (both *p*'s < 0.001), and significantly curved only in recipients (*p* < 0.001) but not in speakers (*p* = 0.877). Taken together, change in speakers' pupil size within utterances is best described by a linear increase from the beginnings to the ends of utterances, while recipients' pupil sizes develop in a curved way within utterances, with a smaller increase at the beginnings of utterances and a plateau in the second half of the utterances (Figure 7).

**Table 4:** Output of linear mixed-effects regression model and *post-hoc* tests on pupil sizes in participants within utterances.

**Fixed effects**				
	β	***SE***	***t***	***p***
Intercept	10.51	3.571	2.967	
Position	5346	360.4	14.835	< 0.001^***^
Position^2^	−1050	364.1	−2.885	< 0.005^**^
Role	0.6246	1.120	0.558	0.577
Size	1.030	0.546	1.886	0.059
Position: Role	5931	720.7	8.229	< 0.001^***^
Position^2^: Role	1584	720.8	2.198	< 0.05^*^
***Post-hoc*** **tests**				
**Contrast**	β	***SE***	***z***	***p***
**Position**				
Speaker	39.3	2.68	14.698	< 0.001^***^
Recipient	11.3	2.11	5.328	< 0.001^***^
**Position** ^2^				
Speaker	−4.27	9.38	−0.455	0.877
Recipient	−30.44	7.43	−4.096	< 0.001^***^

Formula = pupilSize ~ 1 + poly(position_rel, degree = 2) ^*^ role + utteranceSize_scaled + (1 | participant) + (1 | conversation). Asterisks indicate levels of significance: ^*^ indicates *p* < .05; ^**^ indicates *p* < .01; ^***^ indicates *p* < .001.

### 3.3 Correlations of frequency trends and pupil size trends

**Figure 8:**
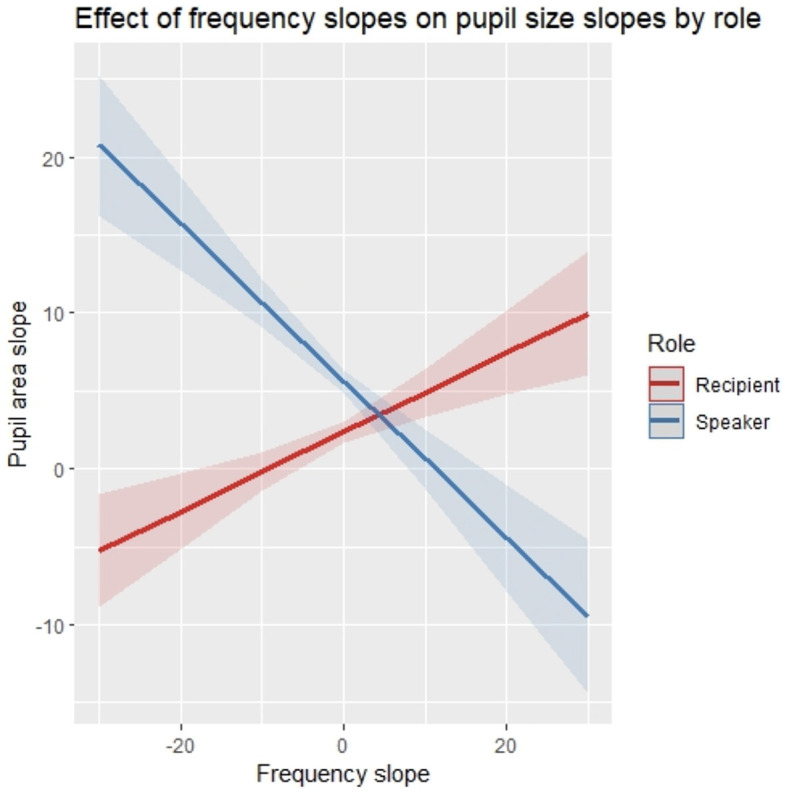
Modeled correlations of slopes in pupil size and slopes in word frequencies within utterances in speakers and recipients. Shaded areas indicate one standard deviation of the mean.

In order to test the relation of trends in frequency and pupil size within turns in speakers and recipients, a mixed-effects model was built, modeling the slope in pupil size within each utterance with the respective slope in word frequencies in the utterance and the role of the participant (speaker vs. recipient) as well as their interaction as fixed effects plus the size of the utterance as an additional control variable in the fixed effects structure of the model. Random intercepts of participant and conversation were modeled as random effects (see Table 5 for model output). The model showed a significant cross-over interaction of frequency slope and role (*p* < 0.001). *Post-hoc* tests controlling for multiple testing found that speakers show a significant inverse relation of pupil size slope and frequency slope (*p* < 0.005) and recipients show a marginally significant direct relation of pupil size slope and frequency slope (*p* = 0.08). This means that the steeper the word frequencies decline from the beginning to the end of an utterance, the bigger the increase in pupil size within that utterance in speakers (and vice versa) but not in listeners, where the relation does marginally point in the other direction (Figure 8). We refrain from interpreting this marginal effect as reliable.

**Table 5:** Modeled pupil size slopes and *post-hoc* tests within utterances in participants.

**Fixed effects**				
	β	***SE***	***t***	***p***
Intercept	3.122	0.607	5.140	
Slope_frequency	−0.124	0.101	−1.225	0.220
Role	3.271	0.743	4.402	< 0.001^***^
Size	−1.613	0.379	−4.255	< 0.001^***^
Slope_frequency: Role	−0.758	0.197	−3.836	< 0.001^***^
***Post-hoc*** **tests**				
**Contrast**	β	***SE***	***z***	***p***
Speaker	−0.504	0.158	−3.200	< 0.005^**^
Recipient	0.254	0.125	2.043	0.080

Formula = slope_pupilSize ~ slope_frequency ^*^ role + utteranceSize_scaled + (1 | participant) + (1 | conversation). Asterisks indicate levels of significance: ^**^ indicates *p* < .01; ^***^ indicates *p* < .001.

## 4 Discussion

Using FreMIC, a novel corpus of free conversation that contains continuous measures of participants' pupil sizes, we tested (i) whether we can attest the systematic pattern of turn-internal frequency distribution showing an S-shaped decline of word frequencies within turns, which was previously observed for written sentences and smaller samples of spoken utterances, (ii) whether speakers' and recipients' pupil size changes within turns show a similar systematicity, and (iii) whether turn-internal frequency distributions are correlated with speakers' and/or recipients' pupil size changes within turns.

We presented three essential findings with regard to these research questions. First, in both the FreMIC and the BNC-C, we found the expected pattern of word frequency distribution within turns-at-talk. In turns of various lengths, turn initial words tend to be highly frequent, turn-medial words tend to be of mid-level frequency, and turn-final words tend to be of much lower frequency again, showing, as in prior research, a frequency decline within turns that follows a three-step staircase pattern, with declines in word frequency after the beginning and before the end of turns. Both drops in word frequency were found to be caused by shifts in the proportions of different word classes across positions in the turn. The early drop in frequencies immediately after turn beginnings was found to be due to a steep decrease of inserts, which have very high frequencies, after turn-initial positions. The late drop in frequencies was found to be caused by a drastic increase in the proportion of content words, especially nouns, which tend to have comparatively low frequencies and which account for the lion share of hapaxes, in turn-final positions. Second, interlocutors' pupil sizes, our operationalized measure for participants' processing load, were found to *increase* as the turn unfolds. Speakers' pupil size in particular was found to increase continuously while the turn-at-talk is being produced. Recipients' pupil size, however, was found to increase much more mildly and tended to not increase any further after the beginning of turns. Third, and notably, the rate of pupil size increases in speakers was found to be correlated to the rate of frequency decrease within the turns-at-talk contained in the corpus. This relation was limited to speakers' pupil size and was not present in recipients' pupil size. These findings have implications for theories of turn structure, information structure, and speech planning in conversational turn-taking. We will discuss each of them in turn.

### 4.1 Turn structure

The vocabulary of the English language is subject to a *drift* dynamic when looked at in its core niche, the turn-at-talk: our findings suggests that while inserts and function words hold sway in turn-early positions, content words, powered by an increase especially in (hapax) nouns, come to prevail in turn-late positions. This provides strong support for Hoey's (2005) theory of lexical priming, especially its textual colligation claim that every word is primed to occur in, or avoid, certain positions of “independently recognized discourse units, e.g., the sentence, the paragraph, *the speech turn*” (Hoey, 2005, p. 115, added emphasis).

The drift dynamic underlying this textual colligation is most obvious in the case of inserts. Their primary job in the turn is to serve as pre-starts (Sacks et al., 1974), that is, to allow the speaker to “begin-with-a-beginning” before the main turn-constructional unit (TCU) is launched. Such pre-starts are drawn from the class of “appositionals”, including inserts such as *well, yeah, oh*, or conjunctions such as *but, and, cos* etc. They count among the top most frequent words in conversation overall (e.g., Rühlemann, 2020a), leading to the commonly observed highly frequent turn beginnings (cf. Heritage, 2015, 2018).

Following (facultative) turn-initial inserts, turns in English often feature a subject in early positions, as English is a subject-verb-object (SVO) language. Together with subject-object-verb (SOV) languages, more than 80% of the languages of the world have the subject as the first canonical constituent (Hammarström, 2016). Theoretically, the subject of a turn—if it has one, since many turns consist of either phrasal or lexical TCUs (Sacks et al., 1974)—can be a noun phrase [with or without an appositional preceding it, as in *Well the back wall looks wet* (BNC-C: KB8) or *The house does get boring* (BNC-C: KCP)].^14^ We presented evidence that the typical subject in conversation is not a noun but a pro-form substituting a noun or noun phrase (or what could be construed as a noun phrase) (cf. Firbas, 1971, 1987, 1996; cf. also Quirk et al., 1985, p. 1356 ff.). Pro-forms are drawn from the class of function words. Like most inserts, pro-forms count among the top most frequent words: *I, you*, and *it* are the three top most frequent words in the BNC-C, *that* (tagged DT0), *he, they, she*, and *we* occupy ranks 9, 11, 13, 21, and 23. A large number of syntactic variants allow speakers to use a pro-form to act as a dummy subject. These variants include not only the afore-mentioned existential-*there* constructions but also other forms of extraposition such as cleft constructions, prop-*it* (*It started to rain* vs. *Rain was starting*) (Quirk et al., 1985, p. 1392), presentational matrix clauses such as *I think, it seems, turns out, the thing is, it's just like* etc. whose communicative purpose lies in acting “as a ‘launching pad' for a new proposition” (Kaltenböck, 2015, p. 118), and the left-dislocation device, as in *This little shop... it's lovely*, where a noun phrase conveying new information (*This little shop*) is separated out from the core of the clause and “replaced” by a co-referential pronoun (*it*), a syntactic device that has a wide currency not only in English conversation (Hughes and McCarthy, 1998, p. 272; Miller and Weinert, 1998, p. 237; Biber et al., 1999, p. 957) but also in many other European languages (e.g., Ashby, 1988). The preference for function-word subjects rather than noun-phrase subjects is also reflected by the fact that virtually the only class of words for which the (English) grammatical system provides pro-forms are nouns [with exception of the pro-verb *do*, whose use is subject to severe syntactic constraints (Seifart et al., 2018)].^15^

Only after the optional pre-start and the pro-form subject do we find the proportion of content words to rise, largely due to a higher proportion of nouns in later positions. While some content words can be highly frequent (e.g., the highest ranking content words in the BNC-C are *know* and *said* ranked 31st and, respectively, 42nd), the vast majority of them are low to extremely low in frequency.

Turns then display a systematic *word-type order* following a pathway from (i) insert (interjections) and/or function word (conjunctions) in the (potential) pre-start position to (ii) function word (pro-forms) in subject position and (iii) (combinations of function word and) content word (most notably nouns) in predicate position. This word-type order is at the same time a frequency as well as an informational order: Pre-starts and pro-forms are low/given information and high-frequency, while content words are high/new information and low-frequency. It is thus relevant to discuss the implications of this order on turn-internal information structure.

### 4.2 Information structure

As found in the presented analyses, turns-at-talk are ordered in terms of the frequency of the words they contain. Relatedly, turns are also ordered in terms of their information structure. They often start with highly frequent inserts, which effectively push the social action to be performed farther into the turn, thus protecting it from overlap (Sacks et al., 1974; Rühlemann and Schweinberger, 2021). In turn-initial position, they act as “harbingers of stance and action in interaction” (Heritage and Sorjonen, 2018, p. 5). Turn-initial *well*, for example, issues a “warning” (Levinson, 1983) that the just-begun turn is not going to align (fully) with the expectations set up in the prior turn (Heritage, 2013, 2015; Rühlemann, 2018). In this way, they can serve a “front-loading bias” (Levinson, 2013, p. 112), adumbrating the action to be implemented in the just-begun turn and thus facilitating recipients' action ascription.

Following (facultative) turn-initial inserts, the core TCU of a turn often contains pro forms in early positions. Clearly, pro-forms have a key role in marking information status, indexing their referents as *given*, that is, as retrievable from context. The pronouns *I, you* and *we* predominantly point to referents immediately available in the situation. Third-person pronouns such as *he, she, it*, and *they* are mostly anaphoric, i.e., co-referential with a referring expression used in prior talk. *It* can be an anticipatory subject as, for example, in cleft constructions such as *it 's him that eats most of the cheese* (BNC-C: KD9) that is cataphorically co-referential with the notional subject. Unattended *this, that, these*, or *those* can be exophoric in reference as in *that's heavy!* (BNC-C: KDN), while unattended *this* and *that* can also be discourse-deictic, referring to an action or an utterance in prior talk as in *Now that's a lie* (Halliday and Hasan, 1976; cf. Levinson, 2004; BNC-C: KCP). Finally, existential *there* is non-phoric, serving merely as a place-holder for the notional subject, as in *There's someone at the door* (BNC-C: KD9; cf. Quirk et al., 1985).

Later turn positions were found to commonly feature a greater number of content words, whose primary function is referential in that they “carry most of the lexical content, in the sense of being able to make reference outside language” (Stubbs, 2002, p. 40), with the referential potential being greatest in nouns (Biber et al., 1999, p. 232). Content words, too, *per se* mark information status: Unlike pro-forms that convey given information, content words are most likely the carriers of new information in the turn. This is especially true for nouns, whose use is “a marked option that is felicitous only in contexts of information novelty, disambiguation needs, or topic and perspective shifts” (Seifart et al., 2018, p. 5721). While, obviously, not every content word is context-independent and hence informationally new, the potential for content words to carry new information is incomparably greater than, say, for function words, which can only carry new information in marked cases (cf. Firbas passim; Quirk et al., 1985, p. 1365 ff.). This assumption is borne out very clearly in the case of content word *hapax legomena*, that is, word types occurring only a single time in the whole corpus: Considering their singularity, they cannot but convey new information, and content word hapax legomena vastly outnumber function word or insert hapax legomena.

The described order underlying the information structure of turns seems to not unreservedly support the Uniform Information Density (UID) hypothesis, according to which informativeness is kept constant at the level of an utterance by smoothing out peaks and dips to “keep the number of bits of information communicated per unit of time approximately constant” (Piantadosi et al., 2011, p. 3526). Given the present findings, that frequency is distributed anticlimactically, while given-new information is distributed climactically, it appears that speakers spread out the information they convey in the turn non-uniformly by increasing informativeness across it rather than keeping it steady. However, we also observed that frequencies remain rather constant in middle positions. Assuming a rough frequency-informativeness correspondence, this would suggest that the UID does hold across turn-internal positions (cf. Klafka and Yurovsky, 2021), which can make up large proportions of longer turns. At the whole-utterance level, however, the present findings suggest that talk in interaction adheres to a “principle of end-focus”, with turns mostly being designed for a “linear presentation from low to high information value” (Quirk et al., 1985, p. 1357). These regularities of “linear modification” have already been noted within the Functional Sentence Perspective developed by the Prague School of Linguistics. Firbas describes linear modification thus: “The closer to the end of the sentence an element comes to stand, the greater the extent to which it contributes toward the development and completion of the communication. Whereas the element occurring finally contributes most to this development, the element occurring initially contributes least to it. Elements occurring neither at the beginning nor at the end rank between the two” (Firbas, 1996, p. 23–24; cf. also Firbas, 1987). So what the information climax suggests for turns-at-talk is an information asymmetry (Prince, 1981, p. 224) extending from low (given) information in turn-early position through intermediate levels of information in turn-middle positions to high (new) information conveyed late in the turn.^16^

### 4.3 Speech production in conversation

A perennial puzzle in psycholinguistics is the apparent ease with which conversationalists manage turn-taking with minimal gaps and overlaps (e.g., Levinson, 2016). Our present findings add to our understanding of the dynamics that help conversationalists achieve such smooth turn-taking.

Turn transitions constitute a crunch zone of speech processing, where next speakers are faced with the dual task of comprehending the incoming turn and planning their response turn (Sacks et al., 1974; Stivers et al., 2009; Levinson and Torreira, 2015; Barthel, 2021). In response to this pressure, and to avoid producing their next turn with a marked delay or losing the right to the next turn completely, next speakers start planning their response turns already during the current speaker's turn (e.g. Barthel et al., 2016, 2017; Bögels et al., 2019), if possible down to phonology (Barthel and Levinson, 2020), even though this strategy has been found to be cognitively demanding (Barthel and Sauppe, 2019). On the other hand, word frequency is particularly relevant for cognitive demand during speech production. Planning high-frequency words is easier, faster, and less error-prone than planning low-frequency words (e.g., Langacker, 1987; Jescheniak and Levelt, 1994; Levelt et al., 1999). That is, to retrieve an infrequent word's morphological, phonological, and phonetic properties places greater cognitive demands on the speaker than to retrieve those of a more frequent word, arguably due to more shallow entrenchment of the former (Langacker, 1987).

This suggests a turn-construction strategy that alleviates processing overload at turn transitions (Barthel and Sauppe, 2019), as planning effort starts low at turn beginnings and increases only as the turn progresses. Firstly, pre-starts benefit the speaker in that they may be produced without having a fully-fledged message ready to be encoded for the upcoming turn and may thus be used to gain planning time close to turn transitions. As pre-starts themselves often contain highly frequent inserts, they are easily retrievable and quickly produced. Speakers thus ease their way into the turn by beginning with a beginning, that is, with one or more easy-to-plan items, like filled pauses or pre-starts. Secondly, and even in the absence of an optional pre-start, speakers commonly produce pro-forms in early turn-positions, often in the form of pro-form subjects. Similar to inserts, pro-forms are highly frequent, commonly phonetically short (cf. Zipf, 1949/1965), and constitute a small number of closed classes with very few members; there are, for example, “only a couple of dozen pronouns” (Stubbs, 2001, p. 40). Thirdly, speakers regularly produce most content words in later positions in the turn, particularly nouns in turn-final position. As these content words tend to be less frequent and phonetically longer (Zipf, 1949/1965; Rühlemann, 2020a), planning and producing them is harder than for inserts and pro-forms (Levelt et al., 1999). The greater retrieval cost for nouns in particular also shows in a robust cross-linguistic tendency for nouns to be produced more slowly than their surrounding speech (Seifart et al., 2018). By placing them in late turn positions, speakers push the phase of high processing load away from the start of the turn, often deep into the turn, possibly as far as until the very end.

Given its quick-and-easy begin-with-a-beginning component, this turn-construction strategy is likely to add to the ease and speed with which turn transitions are managed with minimal gaps and overlaps. In recent descriptions of the psycholinguistic processes of turn-taking in conversation, input prediction and early response planning are the key ingredients to solving the timing puzzle in planning the next turn (Bögels et al., 2015; Gisladottir et al., 2018; Barthel, 2020). Given our findings, however, prediction is only one piece to the puzzle. These models rely on estimates for speech production latencies of around 600 ms for single words (Indefrey and Levelt, 2004; Strijkers and Costa, 2011) and about 1,500 ms for single sentences (Griffin and Bock, 2000; Schnur et al., 2006). These estimates are informed by lab-based experimental settings in picture naming and scene description, respectively. The present findings call into question whether these production latencies are appropriate for modeling natural instances of conversational turn-taking, which commonly contain turn transition times on the order of merely 200 ms (Stivers et al., 2009; Heldner and Edlund, 2010). For one, picture naming experiments and talk-in-interaction are not easily comparable. In a picture naming experiment, the visual object appears on the screen mostly “out of the blue”, without any contextual embedding, whereas in turns-at-talk any prior turn or series of turns provides a rich contextual background against which the current turn is getting produced and interpreted. Prior talk *primes* current talk: The use of a concept in prior talk activates related concepts, raising their activation level and speeding up their retrieval. Moreover, production latency centrally depends on word frequency (next to other factors such as word length) (Levelt et al., 1999; for a more detailed discussion, see Rühlemann, 2020b; for a similar line of reasoning see Knudsen et al., 2020). Based on the observed structural bias of frequent, easy-to-produce words in turn-initial positions [matching the easy-first principle of speech production (MacDonald, 2013)], and less frequent words such as nouns toward late or turn-final positions, turn-initiation latencies are probably greatly reduced in conversational interaction by speakers' active engagement in beneficial turn design. While these findings do not speak against the general assumptions of the consensus model of speech planning in turn-taking, they do fundamentally complement them, reassigning the weights of the relevant sub-tasks of next speakers aiming to produce well-timed turns-at-talk, reducing the importance of content prediction and early response planning and underlining the importance of facilitative turn design.

We find evidence that speakers actually adopt a turn-construction strategy from low to high processing load in that speakers' pupils continuously dilate while they produce the turn: the progressive increase in speakers' processing load during turn production is a reflection of progressively less frequent, harder-to-access words that are planned to be part of the turn-under-construction.^17^ This assumption is supported by the previously observed tendency for speech rate to slow down in the course of turns, an effect referred to as “rallentando”, which affects the turn as a whole (Rühlemann and Gries, 2020).

The high-to-low-frequency turn-construction strategy arguably also has a regulating effect on the distribution of interlocutors' processing load across turns. Any speech exchange system that serves regularly smooth turn transitions needs to be designed to avoid concurrent peaks in processing effort in both speakers and recipients. As one of the solutions to the distribution problem that can be achieved by language evolution, Roberts and Levinson (2017) propose the notion of “end-loading” of information^18^: Facing informationally rich content at the end of an incoming turn does on the one hand lead to a non-trivial comprehension task late in the turn, whereby fast production of a contingent and relevant response turn is made difficult. However, this very problem can on the other hand be alleviated by constructing the response turn following the same pattern of end-loading, also producing the high-information content only at the end of the turn, farthest away from the previous location of high informational density. While Roberts and Levinson assumed verbs to be the critical information-heavy ingredient, our results (based on English corpora) suggest that interlocutors achieve this pattern primarily with turn-final nouns. Either way, the recurrence of the same pattern of frequency distribution within turns can be argued to regulate the distribution of processing load across turns and interlocutors. The attested correlation of the increase in speakers' pupil size and the decrease in frequency in their turns supports the view that much of the processing pressure speakers face in the crunch zone at turn transitions can be reduced when turns start out with highly frequent words and only gradually incorporate less frequent material.

While informationally rich content in turn-final position can be argued to be harder to comprehend than less informative turn-final content, there is nonetheless one additional potential advantage to the end-loading strategy. As low-frequency words will be harder to comprehend than high-frequency words in any position, the lowly frequent, highly informative part at the end of the turn is comparatively well contextualized by the rest of the turn and therefore easier to predict and to integrate into the constructed meaning of the current turn and discourse (Barthel et al., 2024)—an effect regularly exploited in psycholinguistic studies on prediction in language (see Kutas and Federmeier, 2011 for an overview). Moreover, given the observed regular drop in word frequency in turn-final positon, comprehenders can expect such a drop to probably occur at the end of the incoming turn, making it a potential cue for the turn end and thus for an upcoming transition relevance place that comprehenders might successfully orient to in conversation (amongst a large number of other cues, see e.g., Gravano and Hirschberg, 2011; Barthel et al., 2017).

On a final methodological note, by averaging over a large number of cases collected in the FreMIC corpus it was possible to model systematic relations between linguistic events and interlocutors' processing load as indexed by pupil size changes. Yet, pupil size measurements do not offer a fine-grained time resolution, as the latencies of pupil dilation in reaction to cognitive processes can vary in both onset and peak dependent on both situational as well as individual circumstances (Ahern and Beatty, 1979; Beatty, 1982; Beatty and Lucero-Wagoner, 2000; Mathot, 2018; Barthel and Sauppe, 2019). The delay of pupil dilations in connection to cognitive events is presumably a key reason why speakers' pupils can be found to dilate throughout the turns whereas recipients' pupils dilate only initially during turn beginnings and eventually even constrict toward turn ends. Speakers regularly start planning their turns during the previous turn by their interlocutor. Since planning and especially speaking is cognitively more demanding than listening (Kubose et al., 2006; Boiteau et al., 2014), speakers' pupils react more strongly than recipients' pupils to the production of the current turn and are more tightly correlated to the current turn's frequency patterns. By contrast, the initial increase in pupil size in recipients during turn beginnings might at least be partially due to a spill-over effect caused by the delayed pupil dilation that was triggered by the production of the (low-frequency) turn-final word of the previous turn by the recipient.

## 5 Conclusions

This study analyzed masses of naturally occurring utterances “from above”, that is, at a distance from their use in situated talk-in-interaction. While analyses of large corpora inevitably fail to consider myriad details and variations, they do make it possible to discover structures that are hidden to the observer (and the participants) “on the ground”. The structures discovered in this study pertain to the distributions of word frequencies and processing load in turns-at-talk. While frequencies develop anticlimactically, with steep declines after turn beginnings and before turn ends, speakers' processing load increases within turns. Notably, across the large number of analyzed turns, the increase in processing load, measured in the form of increasing pupil size, was found to be inversely correlated to the decline in frequency in the speakers of the turns. This correlation was not attested in recipients.

We propose an interpretation of the observed inverse correlation as an indication that speakers abide by the principle of information climax, seeking to order the information in the turn with a view to maximizing communicative dynamism within the turn. Zipf's *economy of words* thus extends to an *economy of word order*, governing how words are sequentialized within turns-at-talk, favoring a progression from least effort, high-frequency, given-information words to high-effort, low-frequency, new-information content words across the turn. Adopting this turn-construction strategy, speakers address the production bottleneck problem at the conversational crunch zone around turn transitions, as they commonly take over the next turn by producing quickly planned turn-initial words, pushing high-cost, low-frequency words downstream, often as far as the very end of the turn. Additionally, by reserving the prominent turn-final positions for highly informative words, these very words are becoming more predictable for the recipients after the preceding turn-internal context has been laying the ground for them to be processed. Moreover, the turn-final drop in word frequency might be used by next speakers as a lexical cue to turn finality, improving their certainty about the presence of a transition relevance place where they can orderly take over the floor with their next turn.

The corpus linguistic methods of the present analysis give first support to the hypothesis that the underlying cause of the correlation of frequency and processing load contours is related to an information climax in turn structure. To test this hypothesis more directly, much further work is needed, precisely taking into focus what this study has not taken into account: What goes on “on the ground” both in terms of phonology (e.g., where do speakers place the nuclear stress?), semantics (e.g., in how many different semantic contexts do speakers use the words in their turn?; cf. Johns et al., 2012), and interaction (e.g., what course of action are the participants taking?), to name only three necessary components. Additionally, all results reported here can only be claimed to pertain to English. Future research is therefore needed to investigate to what extent the same structures and associations can be found in turns-in-talk in typologically different languages, especially such with different canonical word orders.

Interlocutors in English adapt systematic regularities in turn structure, which are beneficial to efficient information transfer, the organization of turn-taking, and the distribution of processing load in conversation. The present study confirms that frequency impacts cognition in naturally occurring interactive language use. Not only are conversationalists found to have accurate implicit knowledge of the underlying frequency distributions, they also skillfully exploit this knowledge, easing processing and time pressures near turn transitions. Methodologically, this study is among the first to show that pupillometric data from large corpora can be fruitfully mined to reveal systematic patterns in interactional language processing “in the wild” (see also Barthel and Rühlemann, in press).

## Data availability statement

The raw data supporting the conclusions of this article will be made available by the authors, without undue reservation.

## Ethics statement

Ethical approval was not required for the studies involving humans because detailed guidelines were obtained from Freiburg University as to how to structure the consent forms for participants. The studies were conducted in accordance with the local legislation and institutional requirements. The participants provided their written informed consent to participate in this study. Written informed consent was obtained from the individual(s) for the publication of any potentially identifiable images or data included in this article.

## Author contributions

All authors listed have made a substantial, direct, and intellectual contribution to the work and approved it for publication.

## Footnotes

^1^The syllable frequency effect is assumed to be facilitated by speakers' access to a mental syllabary (Levelt and Wheeldon, 1994).

^2^Considerable work by the first author went into associating each word form with its exact PoS tag; this work included, inter alia, weeding out punctuation, correcting misspellings and erroneous PoS tags.

^3^The description of FreMIC that follows will focus exclusively on the aspects and components that are of relevance for the present analyses; for a comprehensive overview see Rühlemann and Ptak (2023). The construction of FreMIC has been facilitated by a grant by the Deutsche Forschungsgemeinschaft (DFG); grant number 497779797.

^4^Other recent multimodal corpora include the Bielefeld Speech and Gesture Alignment Corpus (SaGA; Lücking et al., 2013), which contains 40,000 words and roughly “six thousand gesture units” (Kok, 2017, p. 4) and the InSight Interaction Corpus, consisting of dyadic and triadic interactions in Dutch with exhaustive eyetracking (Brône and Oben, 2015).

^5^The 18 files contain 7 files for dyadic (F02, F03, F05, F06, F09, F11, F13), and 11 files for triadic talk-in-interaction (F01, F04, F07, F08, F12, F15, F16, F18, F19, F20). The total word count is based on c7 tags.

^6^The tagging was done after replacement of unclear speech, silent gestures, comments and the like with placeholder “NA”.

^7^For example, while *c5* distinguishes three categories for conjunctions (CJC for coordination, CJS for subordination, and CJT for *that* used as a conjunction), *c7* has six categories (CC for coordination, CCB for adversative coordination through *but*, CS for subordination, CSN for *than*, CST for *that*, and CSW for *whether*). Another example are pronouns: *c5* has 4 distinct tags, *c7* has 19!

^8^The term “turn” is approximate here, for two reasons. First, the BNC-C only records “utterances”; as shown in Robinson et al. (2022), these are neither 100% identical with TCUs or turns. Second, the unit of observation in FreMIC data is, as noted, the IPU. Again, IPUs may not fully map onto turns. In referring to them as turns nonetheless, we follow Levinson and Torreira (2015) and Levinson (2016), who also based their analyses of *turn*-taking on the IPUs in the Switchboard Corpus.

^9^Clearly, binning pupil data is an *approximate* method as it results in short words such as'*s* or *do* receiving the same amount of pupil measurements as long words such as *marmelade* or *hurly-burly*. Unfortunately, automatic syllabification in the FreMIC is beyond our resources. The focus, however, in the present analysis is not on the cognitive effort spent on particular types of words but on how cognitive effort is distributed and develops across the turn. Binning facilitates a view of that development. Moreover, the vast majority of word tokens in English conversational turns is monosyllabic (cf. Cutler and Carter, 1987). Analyzing a sample of 1,000 10-word turns from the BNC that were phonemically transcribed using Regular Expression for syllabification, we found 82% of the word tokens to be monosyllabic, 14% to be bisyllabic and only 4% to be multisyllabic. Therefore, the method of binning data in equi-space intervals can be considered reasonably good for the present purposes.

^10^The same cubic results pattern was observed for the modeled frequencies in the 3- to 25-word turns in FreMIC.

^11^In the initial positions in turns-at-talk, “well” and also “so” perform a pragmatic function in more than 80% of cases (cf. Rühlemann, 2019). For the above analysis, those initial uses have been re-tagged from adverb to interjection.

^12^Verbs account for 31%, adjectives for 15%, and adverbs for 2% of content word hapaxes.

^13^Drawing on relevant PoS tags, adnominal uses of *this, that, these*, and *those* as well as adverbial uses of *there* were excluded from this analysis.

^14^Quirk et al. (1985, p. 1366) observe that the occurrence of noun phrases in subject position is “especially associated with intransitives” as in *The telephone is ringing* or *A visitor called*.

^15^Pro-verb *do* can be either substitute *do* as in *She likes X. So do I* or a combination of *do* and *it* as in *Then I have to do it*, where *do it* anaphorically refers to some course of action specified in prior talk (Halliday and Hasan, 1976).

^16^In speech, it is assumed that the item carrying the information focus is at the same time the item that carries the intonation nucleus (Quirk et al., 1985). While speakers can choose to place the nucleus on turn-early or turn-middle items, for example for purposes of contrast or correction (Quirk et al., 1985, p. 1365), recent research suggests a right-leaning distribution of the nucleus, overwhelmingly in the second half of the turn (Rühlemann and Schweinberger, 2021).

^17^As noted by an anonymous reviewer, the inverse dilation-frequency association could also partly be explained by “the [speaker's] cognitive effort of tracking (i.e., keeping in memory) an increasingly long utterance that the speaker is building on with each additional word”.

^18^As an early precursor to Roberts and Levinson's notion of “end-loading” note Behaghel's observation that a speaker tends to “place at the end what one particularly wants to imprint on the listener's memory due to its importance” (Behaghel, 1909, p. 138).
